# Highly Efficient Excitonic Recombination of Non-polar ($11\overline{2}0$) GaN Nanocrystals for Visible Light Emitter by Hydride Vapour Phase Epitaxy

**DOI:** 10.1038/s41598-020-58887-7

**Published:** 2020-02-07

**Authors:** Moonsang Lee, Dongyun Lee, Hionsuck Baik, Heejin Kim, Yesul Jeong, Mino Yang, Hyun Uk Lee, Myung Gwan Hahm, Jaekyun Kim

**Affiliations:** 1 Research Center for Materials Analysis, Korea Basic Science Institute, 169-148, Gwahak-ro, Yuseong-Gu Daejeon, 34133 Republic of Korea; 2 Department of Photonics and Nanoelectronics, Hanyang University, Ansan, 15588 Republic of Korea; 3 Seoul Center, Korea Basic Science Institute, 145 Anam-ro, Seongbuk-Gu, Seoul, 02841 Republic of Korea; 4 Busan Center, Korea Basic Science Institute, 60, Gwahaksandan 1-Ro, Gangseo-Gu, Busan, 46742 Republic of Korea; 5 Department of Materials Science and Engineering, Inha University, 100 Inha-Ro, Michuhol-Gu, Incheon, 22212 Republic of Korea

**Keywords:** Materials science, Materials for devices, Materials for optics, Nanoscale materials, Techniques and instrumentation

## Abstract

While non-polar nanostructured-GaN crystals are considered as a prospective material for the realization of futuristic opto-electronic application, the formation of non-polar GaN nanocrystals (NCs) with highly efficient visible emission characteristics remain unquestionable up to now. Here, we report the oxygen-incorporated a-plane GaN NCs with highly visible illumination excitonic recombination characteristics. Epitaxially aligned a-plane NCs with average diameter of 100 nm were formed on r-plane sapphire substrates by hydride vapor phase epitaxy (HVPE), accompanied by the oxygen supply during the growth. X-ray photoemission spectroscopy measurements proved that the NCs exhibited Ga-O bonding in the materials, suggesting the formation of oxidized states in the bandgap. It was found that the NCs emitted the visible luminescence wavelength of 400‒500 nm and 680‒720 nm, which is attributed to the transition from oxygen-induced localized states. Furthermore, time-resolved photoluminescence studies revealed the significant suppression of the quantum confined Stark effect and highly efficient excitonic recombination within GaN NCs. Therefore, we believe that the HVPE non-polar GaN NCs can guide the simple and efficient way toward the nitride-based next-generation nano-photonic devices.

## Introduction

III-Nitride-based nanoscale structures have been gained tremendous attention as candidates suitable for opto-electronic applications, owing to their strong carrier confinement characteristics *via* large band offset between nano materials and matrix, and large exciton binding energy^1–3^. Among these nitride-based materials, gallium nitride (GaN) nanocrystals (NCs) with a direct wide band gap (3.34 eV) extensively have been studied for the use of next generation devices, such as photovoltaic, solid-state quantum computation, and single-photon sources^4–7^. To achieve GaN nanostructures on a substrate, various techniques such as self-assembly growth, vapor-liquid-solid (VLS) process using molecular beam epitaxy (MBE), and metal organic chemical vapor deposition (MOCVD) have been widely used so far^8–10^. For example, Zhang *et al*. reported the formation of GaN NCs *via* thermal decomposition by MOCVD^11^. Additionally, GaN nanodots were grown *via* Ga droplet epitaxy using pretreatment and post-annealing procedure and converted from Ga_2_O_3_ using combustion process^10,12,13^. These methods, however, can exhibit the limited introduction of GaN nanostructures into the desirable applications, because of the complicated growth process and low throughput. In addition, polar GaN with wurzite crystal structure experiences a quantum-confined stark effect (QCSE) along c-axis, which is induced by a large spontaneous polarization field^5^. This leads to a large spatial separation between electron and hole wavefunctions, resulting in a loss of internal quantum efficiency^6,14^. QCSE also causes a long exciton lifetime increasing the time-jitter on the emission from a single photon source and large spectral diffusion, thus deteriorating the performance of the polar GaN-based nanostructure devices^15^.

Since the growth of GaN along non- and semi-polar orientations has been considered as a solution to circumvent these negative effects by eliminating or reducing the internal electric fields^16,17^, it has been of significant interest to the community. However, it is inevitable to dope In or Al elements in non-polar GaN crystals to give rise to the visible luminescence with an acceptable intensity. This can impose the declined luminescence properties of the materials and inefficient fabrication process. Even though several studies have attempted to grow nanostructured-GaN with visible luminescence characteristics using various approaches, such as rare earth doping, and defect-related emission^7,18–20^, the efficient luminescence characteristics was inappropriate for the practical illumination devices.

In this paper, we reported non-polar (11–20) GaN NCs for the application of a highly efficient visible luminescence source by hydride vapour phase epitaxy (HVPE). HVPE, with its high growth rate, could easily grow nanoscale GaN crystals without complicated processes and growth conditions. Furthermore, the introduction of oxygen elements during HVPE growth enabled the localized states within the bandgap to achieve visible light emission with highly efficient excitonic recombination.

## Experimental

### Formation of NCs

**Figure 1:**
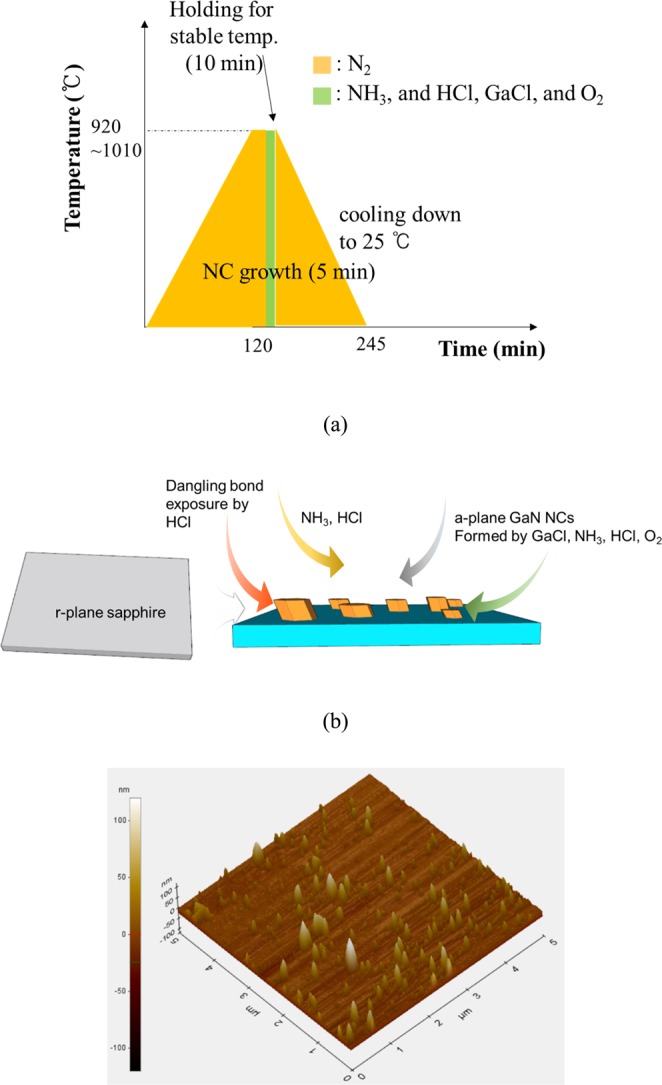
Schematic illustrations of (**a**) growth sequence and (**b**) mechanism of a-plane GaN NCs on r-plane sapphire by HVPE. (**c**) AFM image of the non-polar GaN NCs.

A-plane GaN NCs were grown on 2-inch (1–102) r-plane Al_2_O_3_ substrates (Hi-solar Co., Ltd.), using a vertical-type home-built hot wall HVPE reactor under atmospheric pressure. Firstly, HCl of 20 sccm, and NH_3_ of 300 sccm were supplied into r-plane Al_2_O_3_ substrates in the temperature of 920 °C, respectively. HCl acid gas etched Al_2_O_3_ surface, thus causing oxygen-terminated surface. This facilitated the nucleation of GaN NCs. Simultaneously, to form a-plane GaN nanocrystals with visible luminescence wavelength, O_2_, GaCl, and NH_3_ elements were incorporated into the oxygen-terminated Al_2_O_3_ surface. The gas flow rates of O_2_, HCl, and NH_3_ were 5 sccm, 15 sccm, and 100 sccm, respectively. HCl gas was delivered into Ga metal source zone where HCl and Ga metal reacted, thus forming GaCl. Consequently, this produced oxidized non-polar GaN NCs on r-plane Al_2_O_3_ substrate. The sequence and procedure on the growth of non-polar GaN NCs are detailed in Fig. 1(a,b). This process is similar to other literature except for the intentional oxygen supply^21–23^. The average size and spatial density of the a-plane GaN islands on r-plane sapphire were 100 nm, and 1.5 × 10^9^ cm^−2^, respectively, confirmed by AFM (See Fig. 1(c)). To compare the optical characteristics of non-polar a-plane GaN NCs, oxygen-introduced c-plane GaN NCs with average size of 100 nm were formed on c-plane sapphire substrate.

### Characterization

The size and density of non-polar a-plane NCs were evaluated by atomic force microscopy (AFM, Innova-LabRAM HR800, Horiba Jobin & Bruker). High resolution transmission electron microscopy (HRTEM) analysis was carried out to investigate the structural properties of the NCs using a FEI double Cs corrected Titan3 G2 60–300 S. The elemental mapping of the NCs was obtained using a Super-X detector with an XFEG higher efficiency detection system, which integrates four FEI-designed silicon drifet detectors close to the specimen. Moreover, high resolution X-ray diffraction (XRD, MP-XRD Malvern Panalytical) analysis was conducted to estimate the crystal quality of the non-polar nanomaterials. X-ray photoelectron spectroscopy (XPS, Sigma Probe, Thermo VG Scientific) measurements were employed to confirm the material chemistry after the entire structure formation. The optical properties were determined by photoluminescence (PL) at room temperature using excitation by a He–Cd laser of 325 nm wavelength. In addition, time-resolved PL (TRPL, XperRam S, Nanobase Co., Ltd) measurements were taken at 10 K, using the corresponding monochromater.

## Results and Discussion

**Figure 2:**
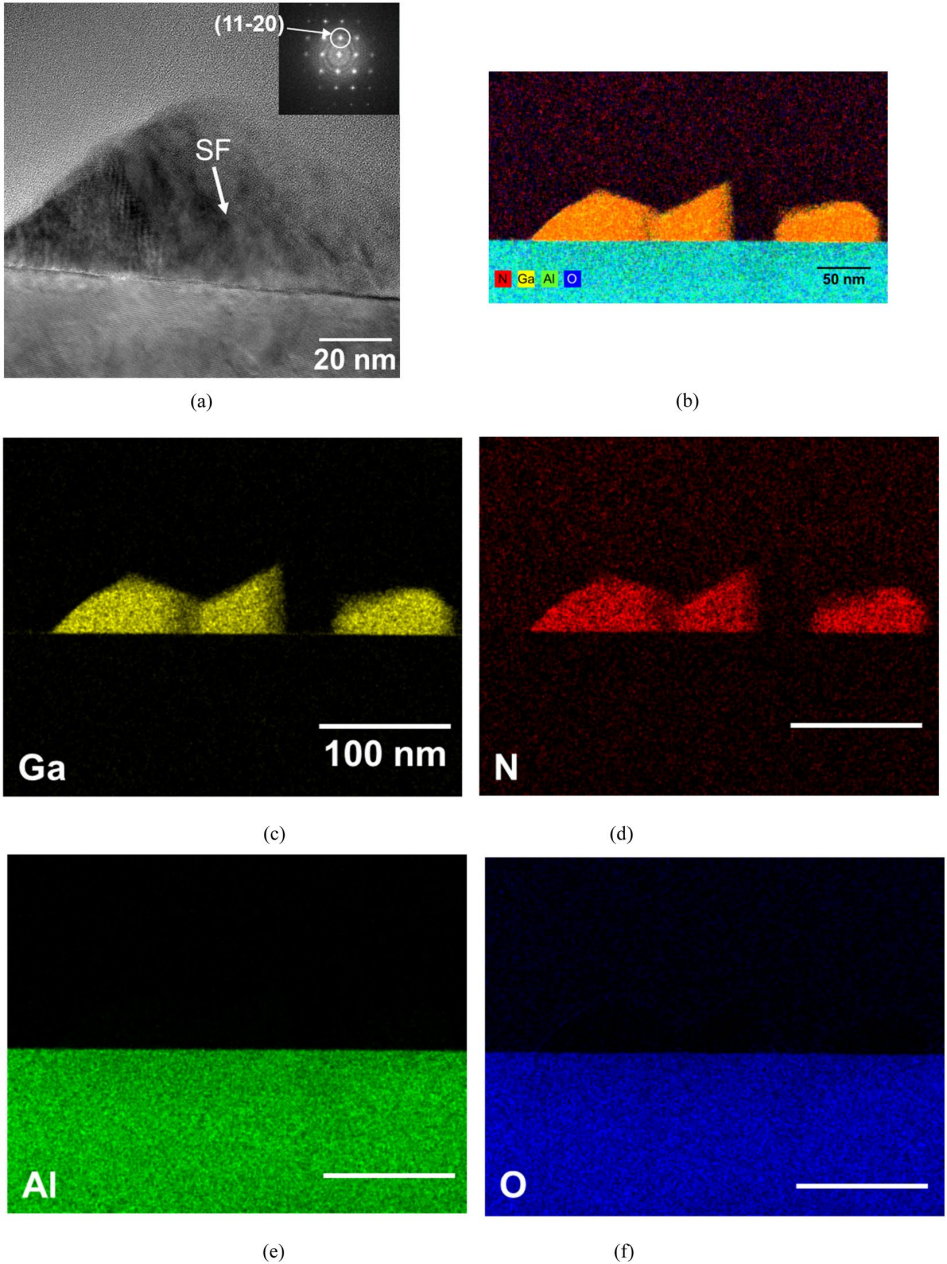
(**a**) Cross-sectional HRTEM image of the non-polar a-plane GaN NCs. The fast Fourier transformation of NC area in the HRTEM image indicates the NC was grown to the direction^11–20^. (**b**) EDS mapping analysis of the corresponding area. EDS elemental analysis of the NCs for (**c**) Ga, (**d**) N, (**e**) Al, and (**f**) O elements did not found any meaningful spectrum related to AlN supposedly to be created by the nitridation on the sapphire substrate^25^.

The TEM analysis shows the non-polar GaN NCs of 100 nm and 60 nm in the average diameter and height, respectively, on the sapphire substrate, as seen in Fig. 2(a). The growth direction of NCs are toward^11–20^ from the fast Fourier transform (FFT) analysis of the high-resolution TEM (HRTEM) image, as shown in the inset of Fig. 2(a). It can be seen that the stacking faults (SFs) are present in the NCs. SF is known as the most typical intrinsic defect in the heteroepitaxial non-polar GaN materials^24^. Notably, an STEM-EDS map confirms that the non-polar GaN NCs consist of the elements of Ga, N, as shown in Fig. 2(b–f). N. Grandjean *et al*. reported the formation of AlN nucleation layer *via* nitridation of an Al_2_O_3_ substrate. According to the literature, an AlN layer was formed on an Al_2_O_3_ surface by exposing the Al_2_O_3_ surface to an NH_3_ gas of 20 sccm at temperature of 850 °C, which was confirmed by *in situ* reflection high-energy electron diffraction^25^. However, almost negligible of Al spectrum on the sapphire substrate by the high temperature surface treatment was observed, suggesting that the NCs were directly deposited on the substrate material. Furthermore, we were not able to detect oxygen elements in the NCs from STEM-EDS map mainly due to the detection limit and oxygen detectability of the EDS equipment, not the absence of the elements in the materials.

**Figure 3:**
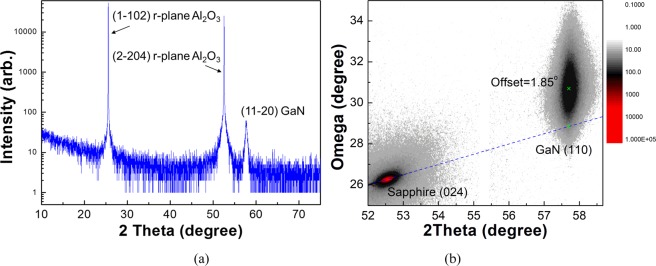
(**a**) XRD theta/2theta scan and (**b**) 2 axis-scan of non-polar a-plane GaN NCs.

To shed light on the crystal orientation and the structure of the GaN NCs, we employed the X-ray diffraction (XRD) analysis, as shown in Fig. 3. We can clearly observe the reflections of r-plane sapphire (1–102), (2, −204) and GaN (11–20), respectively, as shown in Fig. 3(a)^26^. The sharp and narrow peak for (11–20) reflection in GaN NCs supports that the materials exhibit high crystalline. It is noticeable that any additional crystal phases were not detectable except for the original material reflections. This corresponds to STEM-EDP, which also supports that highly aligned pure single crystalline a-plane GaN NCs were successfully grown on r-plane sapphire substrate. Furthermore, 2 axis-scan revealed that the non-polar GaN NCs were deposited on r-plane sapphire with the off-axis of 1.85° along a-axis, as shown in Fig. 3(b).

**Figure 4:**
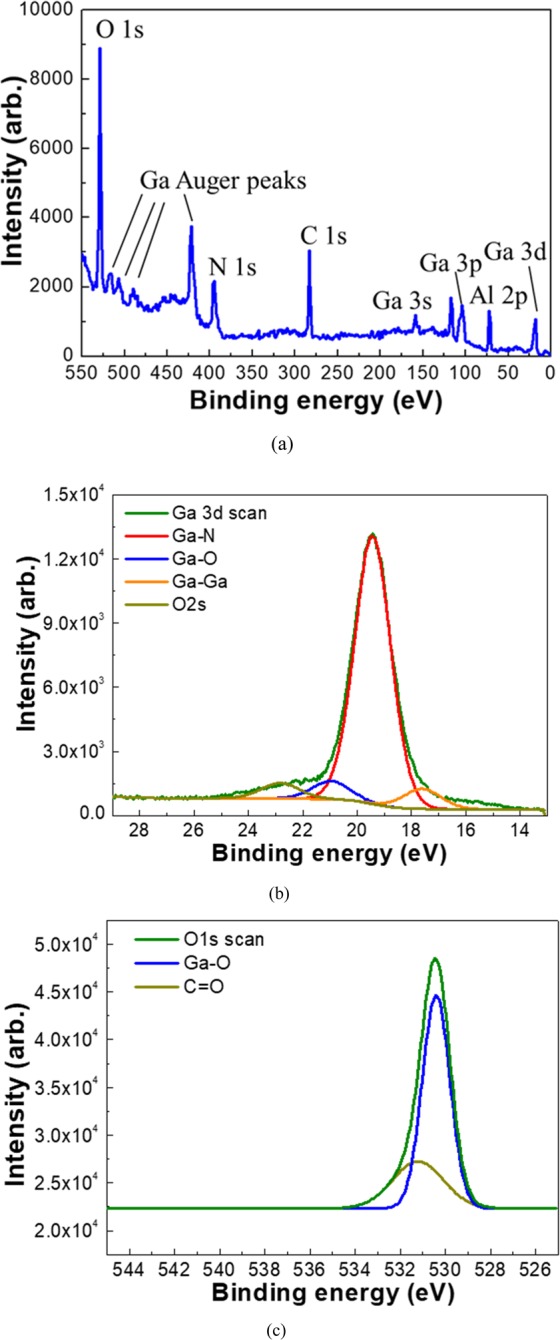
(**a**) XPS survey spectrum of HVPE a-plane GaN NCs. Deconvoluted XPS spectrum of the (**b**) Ga 3d, (**c**) O 1s signal obtained from the non-polar NCs.

To determine the chemical states of the elements in the NC materials, XPS analysis were conducted, as shown in Fig. 4. Figure 4(a) shows XPS survey analysis after the formation of non-polar GaN NCs. Substrate-related Al 2p and O 1 s peaks are apparently positioned at 72.28 eV and 528.21 eV, respectively. Ga-related XPS peaks including Ga Auger peaks, and N 1 s are visible in XPS spectra, confirming the formation of HVPE a-plane GaN NCs on the substrate. This corresponds with the results of STEM-HAADF and XRD. Furthermore, XPS core level spectra investigations for Ga 3d, and O 1 s signals were employed, respectively, as illustrated in Fig. 4(b,c). The deconvoluted Ga 3d core level signals clearly ensure the presence of Ga-N, and Ga-O bonding in the materials, as shown in Fig. 4(b). The signal of Ga-O bonding at 20.9 eV suggests the formation of GaO_x_N_y_ or substitutional oxygen (O_N_) complex defects in the nano-architectures^27^. Since we could not detect the oxide signals in STEM and XRD, we speculate that Ga-O spectrum is associated with the presence of O_N_-related point defect complex. In addition, Ga-O bond peak centered at 530.5 eV in O1s core level spectra further provided that the nature of GaN NCs involved oxygen elements, as illustrated in Fig. 4(c)^28^.

**Figure 5:**
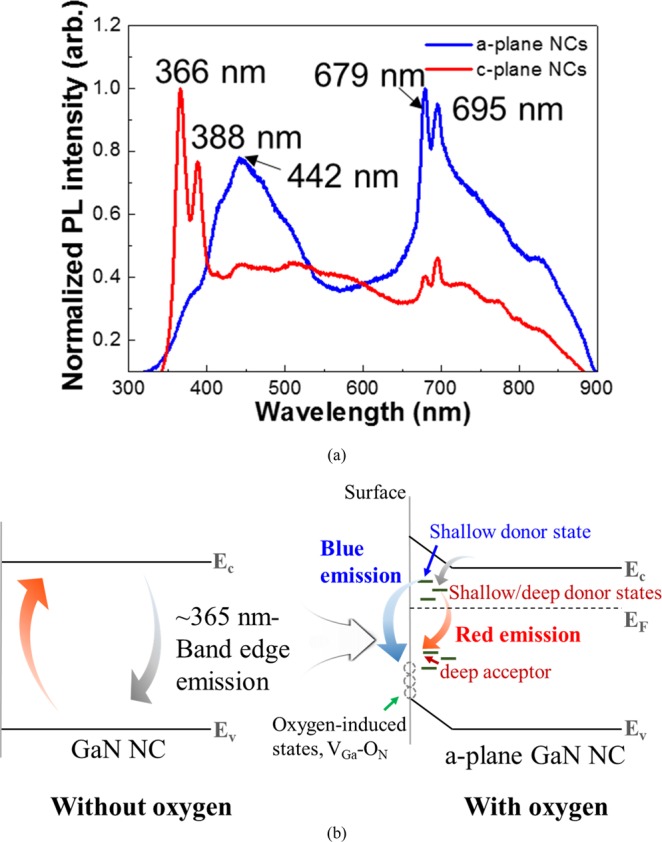
(**a**) PL spectra of c- (red), and a-plane (blue) GaN NCs with 100 nm-diameter at room temperature. (**b**) Schematic illustration of bleached photoluminescence of HVPE a-plane GaN NCs.

To investigate the optical characteristics of HVPE a-plane GaN NCs, we performed PL measurements, as presented in Fig. 5(a). Typically PL spectrum of c-plane GaN layers on sapphire substrates shows band edge emission at 365 nm and yellow luminescence band^29^. However, c-plane NCs exhibits dominant wavelength at 366 nm and 388 nm, accompanied with broad plateau in the wavelength range from 400‒800 nm. We consider that carbon- and oxygen-related defect complex imposes the broad red luminescence in c-plane GaN nanodots^30–32^. The 366 nm peak close to the bandgap energy of GaN can be assigned to exciton recombination. It is well known that 388 nm wavelength in GaN materials is related to structural defects, such as stacking faults and screw dislocation along c-axis^33–35^. On the sharp contrary, we can clearly observe that the dominant peaks of a-plane GaN NCs appeared around blue and red emission spectra of 400‒500 nm and ~680 nm. The peak positions of the NCs were centered at wavelength of 442 nm and 680 nm. The broad shape of PL peaks may be related to the size distribution of GaN NCs and defect-related states. One can observe that near band edge (NBE) emission around 366 nm in a-plane GaN NCs is present but very weak. It is noticeable that structural defect-related emission at the wavelength of 388 nm is also declined in PL spectrum of non-polar GaN NCs, compared to that of c-plane ones. Since it is well established that the densities of extended defects in non-polar GaN crystals are much higher than those of c-plane ones^24^, we consider that this is not attributed to the extended structural defects in non-polar nanomaterials. Rather, we assign that this nature originated visible luminescence transition produced from defect-induced deep level states in the bandgap of the NCs due to the oxygen influx during the HVPE growth. Note that the emission energy is smaller than the energy of GaN band edge recombination, associated with the presence of the quantum confinement Stark effect, inherited from the internal electric field in the structure^36^. Since non-polar materials exhibit the negligible piezoelectric field, we speculate that this can be related to the electronic deep trap states in the NCs, such as donor and acceptor pair (DAP), Mg-, carbon-, and intrinsic-related defects^37–41^. Reshchikov *et al*. reported that the subsequent oxidation of GaN materials would evolve the blue band in the GaN, which originated from a transition from the shallow donors in the near surface region to band-bended surface states^42^. In other words, luminescence between the electrons captured in shallow donors and the holes localized by the oxidized surface states can emit the blue emission of GaN materials. Additionally, it has been reported that red luminescence of GaN reflects the transition from a shallow/deep donor or from the conduction band to an unknown deep acceptor, inherited from the presence of V_N_C_N_ or V_Ga_O_N_^30–32^. Note that HVPE growth of GaN NCs was performed with O_2_ supply, implying the introduction of oxidized states in the materials. Indeed, even if the flow rate of O_2_ during a-plane GaN NC growth is very low, typical HVPE GaN epitaxial layers exhibits more than oxygen concentration of 10^16^/cm^3^, which can also help the formation of the defect-related states in GaN materials^43,44^. Since impurities like C, or Mg were not used in this case, consequently, we consider that the significant incorporation of oxygen in the NCs take part in the reformation of the visible blue and red luminescence of a-plane GaN NCs, as shown in Fig. 5(b). The presence of the oxygen peak in XPS result of Fig. 3 further support this statement. V. Jindal *et al*. computed the surface energies as a function of crystallographic orientations of GaN^45^. They addressed that the surface energies of a-plane GaN, and Ga-face c-plane GaN were estimated to be 159 meV/Å^2^, and 129 meV/Å^2^, respectively. We assure that this is attributed to higher surface energy of a-plane GaN. It is well established that the higher surface energy, the higher surface unstability, thus encouraging much higher reaction with other materials^46^. This clearly implies that the reaction with supplied O_2_ elements could be much activated in a-plane GaN with higher surface energy, compared to Ga-face c-plane GaN, thus making higher oxidized states of a-plane GaN NCs. Consequently, we speculate that this results in the nature of broad visible luminescence range in a-plane GaN NCs. To achieve visible luminescence in GaN-based devices, it is essential to incorporate dopant materials, such as indium (In) elements in GaN crystals. This can cause the piezoelectric fields in the materials, thus reducing the efficiency of device by the separation of hole and electron wave-functions^47^. However, we believe that oxygen-mediated a-plane GaN nanocrystal materials on n-GaN layer/sapphire substrates can be easily utilized as visible luminescence photonic devices only by inserting p-GaN layer. The device is under study. One can found the co-existence of 679 nm and 695 nm peak position in c- and a-plane GaN. It is well established that luminescence of 679 nm is related to high concentrations of C and O, and the overlap of the donor acceptor pair (DAP) band and the electron-to-acceptor (e,A0) transition band. Furthermore, emission at 695 nm is attributed to DAP transition^31,32^.

**Figure 6:**
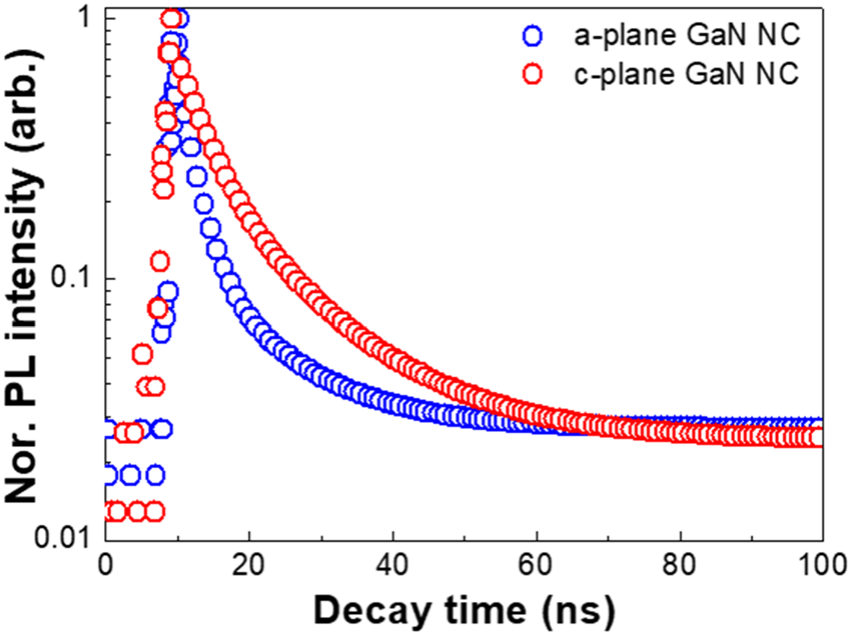
Time-resolved PL spectra of oxygen-induced a- and c-plane HVPE GaN NCs with different sizes at 10 K. The blue and red line represent the PL decay curves of a-plane GaN NCs and c-plane GaN NCs, respectively.

In order to clarify the carrier characteristics in a-plane GaN NCs, TRPL measurements were carried out at 442 nm emission peak and 10 K by estimating the lifetime of excitons in a-plane and c-plane HVPE GaN NCs with the corresponding NC sizes of 100 nm, as seen in Fig. 6. The PL lifetime curves are well fitted with double exponential functions as follows:

1 $${\rm{I}}({\rm{t}})={{\rm{A}}}_{{\rm{fast}}}\exp (-{\rm{t}}/{{\rm{\tau }}}_{{\rm{fast}}})+{{\rm{A}}}_{{\rm{slow}}}\exp (-{\rm{t}}/{{\rm{\tau }}}_{{\rm{slow}}})$$

where A_fast_ and A_slow_ are the normalization constants, t is time, τ is the photo-excited carrier lifetime, and I(t) is the time-dependent PL intensity. All the parameters in the double exponential function are presented in Table 1.

**Table 1:** TRPL parameters extracted from fitting of the double exponential function for a-plane GaN NCs, and c-plane GaN NCs.

	τ_fast_ (ns)	τ_slow_ (ns)
a-plane NCs	0.44	5.7
c-plane NCs	5.38	10.81

It is noticeable that the all measured decay time for the c-plane GaN NCs shows much longer lifetimes than that for a-plane GaN ones. The fast decay regions implies the fast exciton recombination process. On the other hand, the localized carrier recombination process dominates the slow decay components^48^. Since TRPL measurements were conducted at low temperature, non-radiative recombination in NCs is negligible. Therefore, we consider that the shorter exciton recombination time of a-plane HVPE GaN NCs is attributed to a significantly increased overlap of electron and hole wavefunctions and reduction of the internal electric filed in HVPE a-plane GaN NCs, indicative of highly enhanced excitonic recombination^49^. This is in excellent agreement with previous results reported elsewhere^6,50–52^. We believe that these remarkably improved optical properties and simple formation of HVPE non-polar a-plane GaN NCs prove to be a viable matrix for a direct visible emission source.

## Conclusions

It was demonstrated that HVPE non-polar a-plane GaN NCs with oxygen-mediated localized states were grown on r-plane sapphire substrates for direct visible light emission. TEM, XRD analysis showed that the formation of the NCs were epitaxially deposited along a-axis of 1.85° on r-plane Al_2_O_3_ structures. XPS analysis proved that the nature a-plane GaN NCs is comprised of Ga-N bonding and O_N_ point defect complex, indicative of the presence of oxygen-induced localized states in the bandgap. Photoluminescence studies also confirmed the blue- and red-light emission of the NCs around wavelength of 400‒500 nm, and 680‒720 nm with the reduced NBE and structural defect-related spectrum, compared to those of c-plane ones. These visible emission bands are attributed to the localized states of GaN NCs, inherited from their oxidized phases. Furthermore, the relatively shorter lifetime of the NCs in TRPL analysis suggested the suppression of the internal electric field and enhanced efficient excitonic recombination. Therefore, this study could offer a new feasible approach to achieve GaN NCs for direct visible luminescence application.

## References

[1] Kabi S, Perera AU (2015). Effect of quantum dot size and size distribution on the intersublevel transitions and absorption coefficients of III-V semiconductor quantum dot. J. Appl. Phys..

[2] Huber D (2017). Highly indistinguishable and strongly entangled photons from symmetric GaAs quantum dots. Nat. Commun..

[3] Yu J (2016). Study on spin and optical polarization in a coupled InGaN/GaN quantum well and quantum dots structure. Sci. Rep..

[4] Yang W (2014). High density GaN/AlN quantum dots for deep UV LED with high quantum efficiency and temperature stability. Sci. Rep..

[5] Hui X (2013). Fabrication of GaN nanodots via GaN thermal decomposition in H2 atmosphere. J. Vac. Sci. Technol. B..

[6] Zhu T (2013). Non-polar (11–20) InGaN quantum dots with short exciton lifetimes grown by metal-organic vapor phase epitaxy. Appl. Phys. Lett..

[7] Saron K, Hashim M (2013). Broad visible emission from GaN nanowires grown on n-Si (1 1 1) substrate by PVD for solar cell application. Superlattices Microstruct..

[8] Hu C-W, Bell A, Ponce F, Smith D, Tsong I (2002). Growth of self-assembled GaN quantum dots via the vapor–liquid–solid mechanism. Appl. Phys. Lett..

[9] Kondo T, Saitoh K, Yamamoto Y, Maruyama T, Naritsuka S (2006). Fabrication of GaN dot structures on Si substrates by droplet epitaxy. phys. stat. sol.(a).

[10] Yu S (2014). Characterization and density control of GaN nanodots on Si (111) by droplet epitaxy using plasma-assisted molecular beam epitaxy. Nanoscale Res. Lett..

[11] Zhang J (2014). Fabrication of low-density GaN/AlN quantum dots via GaN thermal decomposition in MOCVD. Nanoscale Res. Lett..

[12] Qi Z (2015). Influence of high-temperature postgrowth annealing under different ambience on GaN quantum dots grown via Ga droplet epitaxy. Opt. Mater. Express.

[13] Chen Y, Jyoti N, Kim J (2011). Strong deep-UV and visible luminescence from GaN nanoparticles. Appl. Phys. A.

[14] Griffiths JT (2014). Growth of non-polar (11–20) InGaN quantum dots by metal organic vapour phase epitaxy using a two temperature method. APL materials.

[15] Ostapenko IA (2010). Large internal dipole moment in InGaN/GaN quantum dots. Appl. Phys. Lett..

[16] Das A (2011). Improved luminescence and thermal stability of semipolar (11–22) InGaN quantum dots. Appl. Phys. Lett..

[17] Feng S-W, Tu L-W, Wang H-C, Sun Q, Han J (2012). The role of growth-pressure on the determination of anisotropy properties in nonpolar m-plane. GaN. ECS J. Solid State Sci. Technol..

[18] Saleem U (2017). Yellow and green luminescence in single-crystal Ge-catalyzed GaN nanowires grown by low pressure chemical vapor deposition. Opt. Mater. Express.

[19] Pan X, Zhang Z, Jia L, Li H, Xie E (2008). Room temperature visible green luminescence from a-GaN: Er film deposited by DC magnetron sputtering. J. Alloys Compd..

[20] Mitchell B (2016). Utilization of native oxygen in Eu (RE)-doped GaN for enabling device compatibility in optoelectronic applications. Sci. Rep..

[21] Lee, M., Vu, T. K. O., Lee, K. S., Kim, E. K. & Park, S. Electronic Transport Mechanism for Schottky Diodes Formed by Au/HVPE a-Plane GaN Templates Grown via *In Situ* GaN Nanodot Formation. *Nanomaterials* **8** (2018). 10.3390/nano8060397 PMC6027380 29865230

[22] Lee M, Mikulik D, Park S (2017). Thick GaN growth via GaN nanodot formation by HVPE. Cryst. Eng. Comm..

[23] Lee M, Yang M, Wi J-S, Park S (2018). Formation of *in situ* HVPE a-plane GaN nanodots: effects on the structural properties of a-plane GaN templates. Cryst. Eng. Comm..

[24] Craven M, Lim S, Wu F, Speck J, DenBaars S (2002). Structural characterization of nonpolar (1120) a-plane GaN thin films grown on (1102) r-plane sapphire. Appl. Phys. Lett..

[25] Grandjean N, Massies J, Leroux M (1996). Nitridation of sapphire. Effect on the optical properties of GaN epitaxial overlayers. Appl. Phys. Lett..

[26] Huang H-M (2012). Growth and Characteristics of a-Plane GaN on ZnO Heterostructure. J. Electrochem. Soc..

[27] Li D (2001). Selective etching of GaN polar surface in potassium hydroxide solution studied by x-ray photoelectron spectroscopy. J. Appl. Phys..

[28] Guzmán G, Herrera M, Silva R, Vásquez G, Maestre D (2016). Influence of oxygen incorporation on the defect structure of GaN microrods and nanowires. An XPS and CL study. Semicond. Sci. Technol..

[29] Calleja E (1997). Yellow luminescence and related deep states in undoped GaN. Phys. Rev. B.

[30] Reshchikov MA, Usikov A, Helava H, Makarov Y (2014). Fine structure of the red luminescence band in undoped GaN. Appl. Phys. Lett..

[31] Wang L, Richter E, Weyers M (2007). Red luminescence from freestanding GaN grown on LiAlO2 substrate by hydride vapor phase epitaxy. phys. stat. sol.(a).

[32] Wang L (2006). Characterization of free standing GaN grown by HVPE on a LiAlO2 substrate. phys. stat. sol.(a).

[33] Elsner J (1998). Deep acceptors trapped at threading-edge dislocations in GaN. Phys. Rev. B.

[34] Ravash R (2009). Metal organic vapor phase epitaxy growth of single crystalline GaN on planar Si (211) substrates. Appl. Phys. Lett..

[35] Yang Y (1999). Blue luminescence from amorphous GaN nanoparticles synthesized *in situ* in a polymer. Appl. Phys. Lett..

[36] Leroux M (1998). Quantum confined Stark effect due to built-in internal polarization fields in (Al, Ga) N/GaN quantum wells. Phys. Rev. B.

[37] Li S (2004). Study of the blue luminescence in unintentional doped GaN films grown by MOCVD. J. Lumin..

[38] Chung S, Suh E-K, Lee H, Mao H, Park S (2002). Photoluminescence and photocurrent studies of p-type GaN with various thermal treatments. J. Cryst. Growth.

[39] Santana G (2013). Photoluminescence study of gallium nitride thin films obtained by infrared close space vapor transport. Materials.

[40] Gruzintsev A, Kaiser U, Khodos I, Richter W (2001). Fine structure of the blue photoluminescence in high-purity hexagonal GaN films. Inorg. Mater..

[41] Teisseyre H (2000). Different character of the donor-acceptor pair-related 3.27 eV band and blue photoluminescence in Mg-doped GaN. Hydrostatic pressure studies. Phys. Rev. B.

[42] Reshchikov MA, Visconti P, Morkoç H (2001). Blue photoluminescence activated by surface states in GaN grown by molecular beam epitaxy. Appl. Phys. Lett..

[43] Khromov S, Hemmingsson C, Monemar B, Hultman L, Pozina G (2014). Optical properties of C-doped bulk GaN wafers grown by halide vapor phase epitaxy. J. Appl. Phys..

[44] Iwinska M (2016). Homoepitaxial growth of HVPE-GaN doped with Si. J. Cryst. Growth.

[45] Jindal V, Shahedipour-Sandvik F (2009). Theoretical prediction of GaN nanostructure equilibrium and nonequilibrium shapes. J. Appl. Phys..

[46] Lee M (2014). Effect of additional hydrochloric acid flow on the growth of non-polar a-plane GaN layers on r-plane sapphire by hydride vapor-phase epitaxy. J. Cryst. Growth.

[47] Gong Z (2011). Electrical, spectral and optical performance of yellow–green and amber micro-pixelated InGaN light-emitting diodes. Semicond. Sci. Technol..

[48] Choi C (2001). Time-resolved photoluminescence of In x Ga 1− x N/G a N multiple quantum well structures: Effect of Si doping in the barriers. Phys. Rev. B.

[49] Schulz S, O’Reilly EP (2011). Built-in field reduction in InGaN/GaN quantum dot molecules. Appl. Phys. Lett..

[50] Taylor R (2004). Dynamics of single InGaN quantum dots. Physica E Low Demins. Syst. Nanostruct..

[51] Robinson JW (2003). Time-resolved dynamics in single InGaN quantum dots. Appl. Phys. Lett..

[52] Reid B (2014). High temperature stability in non‐polar (11$\bar 2 $0) InGaN quantum dots: Exciton and biexciton dynamics. physica status solidi (c).

